# Long-term optical brain imaging in live adult fruit flies

**DOI:** 10.1038/s41467-018-02873-1

**Published:** 2018-02-28

**Authors:** Cheng Huang, Jessica R. Maxey, Supriyo Sinha, Joan Savall, Yiyang Gong, Mark J. Schnitzer

**Affiliations:** 1 James H. Clark Center, Stanford University, Stanford, CA 94305 USA; 2 CNC Program, Stanford University, Stanford, CA 94305 USA; 3 Howard Hughes Medical Institute, Stanford University, Stanford, CA 94305 USA; 4 Department of Biomedical Engineering, Duke University, Durham, NC 27708 USA

## Abstract

Time-lapse in vivo microscopy studies of cellular morphology and physiology are crucial toward understanding brain function but have been infeasible in the fruit fly, a key model species. Here we use laser microsurgery to create a chronic fly preparation for repeated imaging of neural architecture and dynamics for up to 50 days. In fly mushroom body neurons, we track axonal boutons for 10 days and record odor-evoked calcium transients over 7 weeks. Further, by using voltage imaging to resolve individual action potentials, we monitor spiking plasticity in dopamine neurons of flies undergoing mechanical stress. After 24 h of stress, PPL1-α’3 but not PPL1-α’2α2 dopamine neurons have elevated spike rates. Overall, our chronic preparation is compatible with a broad range of optical techniques and enables longitudinal studies of many biological questions that could not be addressed before in live flies.

## Abstract (web summary)

Time-lapse imaging studies of more than a day in the fly brain have been infeasible until now. Here the authors present a laser microsurgery approach to create a permanent window in the fly cuticle to enable time-lapse imaging of neural architecture and dynamics for up to 10–50 days.

## Introduction

Direct and repeated observations of cellular structure and physiology over extended periods in live animals are critical to dissecting the mechanisms of experience-dependent plasticity, neural development, degeneration, and aging^1^. Chronic preparations for long-term intravital microscopy exist for several animal model species that are either naturally translucent or allow installation of optical windows. Time-lapse studies using these preparations have revealed rich longitudinal dynamics for cellular morphology and function, under both natural and pathological conditions, that could not be captured by studying multiple distinct sets of animals at different time points^2–4^.

**Fig. 1:**
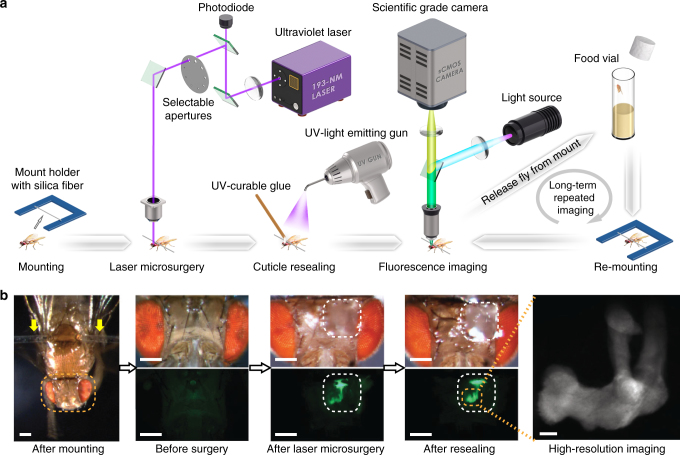
Laser surgical preparation of live flies for long-term in vivo microscopy. **a** Workflow for repeated, long-term in vivo imaging in the fly brain. We first mounted the fly onto a silica fiber, which was attached to the thorax (Supplementary Movie 1). We then used a laser microsurgery system, based on a 193-nm-wavelength excimer laser^6^, to cut an observation window in the cuticle of the mounted fly. We resealed the opening in the cuticle with ultraviolet-light-cured transparent epoxy. We held the mounted fly under the objective lens of a fluorescence microscope for in vivo brain imaging. At the end of each imaging session, we detached the fly from its silica fiber mount and placed the fly in a food vial for long-term storage. To conduct subsequent imaging sessions, we re-mounted the fly on a silica fiber, performed in vivo fluorescence brain imaging, and re-released the fly into a food vial. In this way, we performed multiple imaging sessions per fly, over time spans of up to 50 days. **b** Steps to creating a transparent window in the fly cuticle by laser microsurgery, along with example images. Left, The fly is mounted using a silica fiber (yellow arrows) attached to the thorax. Three middle panels, Bright field images (top row) of the fly head before and after surgery, and then after the laser-cut window in the cuticle (250 × 200 μm^2^; area enclosed by dashed line) is filled with transparent, UV-light curable glue. Fluorescence images (bottom row) taken at each stage of the procedure show the optical access gained to GFP-expressing neurons in the mushroom body of a female *OK107-GAL4>20×UAS-6×GFP* fly. Right, A postsurgical, two-photon image of the mushroom body lobe acquired through the imaging window. The image shown is a mean intensity projection, averaged over a three-dimensional stack of 33 images, acquired 3 μm apart in the axial dimension. All scale bars are 200 μm, except the rightmost scale bar, which is 20 μm

In the fruit fly, a mainstay animal model for biological research, long-term in vivo imaging (>24 h) of the intact central nervous system has remained elusive due to the fly’s fragility and opaque exoskeleton^5, 6^. Hence, many questions remain unanswered about how neural circuits develop, respond to experience or environmental influences, or degenerate due to injury or disease. To facilitate time-lapse studies of these issues, we developed a workflow for repeated, long-term in vivo imaging in the fly brain for up to 50 days (Fig. 1a). As we illustrate here with several types of time-lapse imaging experiments, this workflow permits longitudinal studies of many different biological questions that could not be examined before in live flies.

## Results

### Laser surgery for long-term optical access to the fly brain

First, we created a detachable mount that can hold an alert fly under a microscope objective lens by affixation of a silica fiber to the thorax (Fig. 1a, b; Supplementary Movie 1). When held this way, a fly’s antennae are free for studies of sensory neurophysiology (Supplementary Fig. 1a–d). Once a fly was mounted, we placed it under the objective lens of a custom-built laser microsurgery system, based on a 193-nm-wavelength multimode excimer laser^6^. Using this microsurgery system, we opened an observation window in the cuticle of the fly’s head.

Excimer-laser-based microsurgery has submicron cutting precision^6^, more than sufficient for creating the 150–250 μm diameter windows we made in the cuticle. However, when applied in isolation, laser microsurgery only allows in vivo brain imaging over 6–18 h time spans^6^. To enable long-term imaging studies, after creating the observation window we resealed the cuticle opening with transparent epoxy (Fig. 1a, b). After surgery, flies moved their legs and wings well, an indicator of postsurgical health. Surgically created windows in the fly cuticle enabled high-resolution fluorescence imaging, which we first demonstrated by imaging neuronal structures within the fly mushroom body (Fig. 1b; Supplementary Fig. 1e, f).

At the end of a surgery or imaging session, we released the fly from the mount, detached the silica fiber from the thorax, and transferred the fly to a food vial for long-term storage. To conduct subsequent imaging sessions, we iterated the mounting, imaging, and releasing steps and thereby performed repeated imaging bouts over extended time spans (Fig. 1a). With practice, we were able to perform surgery on eight flies per hour, at a surgical success rate of ~85% for flies 2–10 day post-eclosion and ~70% for flies <8 h post-eclosion.

### Surgery minimally affects female fly lifespan

**Fig. 2:**
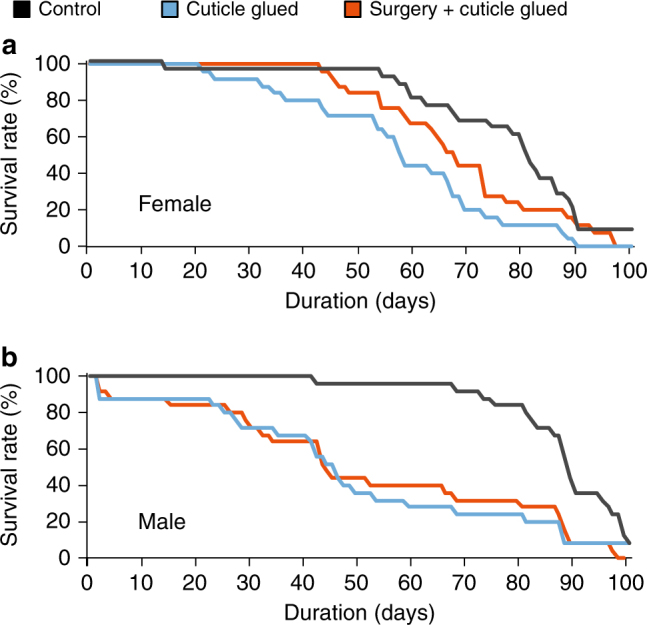
Survival curves of male and female flies after laser microsurgery. **a** The lifespans of female flies that underwent the full surgical procedure (laser microsurgery plus glue atop the optical window) were statistically indistinguishable from those of control flies (*P* = 0.2; *n* = 25 flies per group; log-rank test) or flies that received glue on the cuticle but no surgery (*P *= 0.06; *n* = 25 flies per group; log-rank test). **b** Male flies that received either the full surgical procedure or glue on the cuticle had reduced lifespans in comparison to control flies that received neither procedure (*P* = 1.2 × 10^−4^ and *P *= 4.3 × 10^−4^; *n* = 25 flies per group; log-rank test)

To gauge the impact of surgery in more detail, we compared the lifespans of untreated control flies against those of flies that underwent either surgery and gluing of the cuticle (surgery group) or only gluing of the cuticle (glue group). Notably, female flies in the surgery and glue groups had a 100% survival rate at 10 days after surgery (*n* = 25 flies per group), and a few flies lived for up to 90 days (Fig. 2a). The lifespan curve of female flies in the surgery group was similar to that of the control group (*P* = 0.2; *n* = 25 flies per group; log-rank test) and the glue group (*P* = 0.06).

By comparison, male flies had a ~90% survival rate 10 days after surgery, and as with females, a few males persisted for ~90 days (Fig. 2b). Lifespan curves of male flies in the surgery and glue groups differed from that of control flies (*P* = 1.2 × 10^–4^ and 4.3 × 10^–4^, respectively, *n* = 25 flies per group; log-rank test; Fig. 2b). Although survival for 10 days in male flies is ~10–100 times longer than what prior surgical techniques have allowed, to extend our studies to the maximum possible durations we used female flies for all subsequent experiments.

### Limited surgical effects on fly behavior

**Fig. 3:**
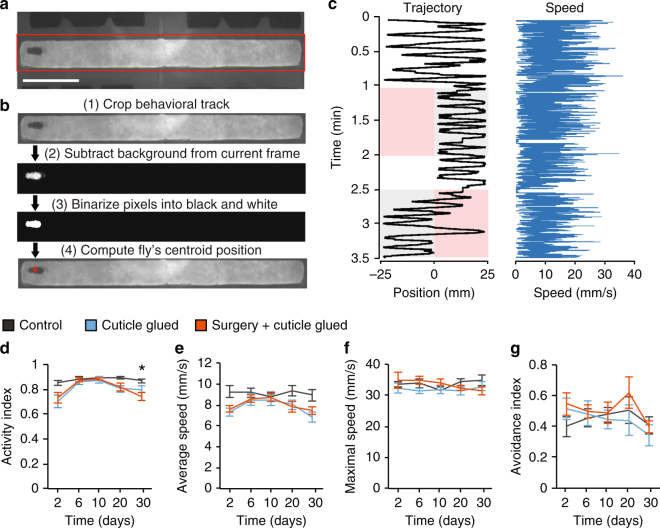
Virtually normal locomotor and odor avoidance behaviors for 30 days after surgery. **a** Behavioral chamber for locomotor and odor avoidance assays, with odor delivery inlets at both sides. Red box: image region-of-interest analyzed to track fly movements. Scale bar: 10 mm. **b** Fly tracking steps: (1) Isolate the region-of-interest; (2) Subtract the mean background image; (3) Group pixels into background (black) and foreground/object (white) pixels using Otsu’s method and filter objects by size to isolate the fly; (4) Compute fly’s current centroid. **c** An example of walking trajectory (left) and speed trace (right). Repulsive odor, 4-methyl-cyclohexanol (3% in air), entered first from the left and then the right inlet. Pink indicates these intervals and entry routes; the opposite inlet delivered fresh air (gray shading). Otherwise, both inlets delivered air (white). **d** Flies that had the full surgery or glue on the cuticle had indistinguishable activity indices from control flies on Days 6 (*P* = 0.9) and 10 (*P* = 0.6) (Kruskal–Wallis ANOVA; 10 flies per group). On Days 2 (*P* = 0.03) and 20 (*P* = 0.04), the groups exhibited significant differences (*n* = 8–10 flies per group; Kruskal–Wallis ANOVA), which were insufficient to yield significant paired post-hoc tests between groups (*P* = 0.06–0.9; *U*-test with Holm–Bonferroni correction). On Day 30, Kruskal–Wallis ANOVA revealed significant differences between groups (*P* = 0.02, *n* = 7–10 flies); post-hoc testing showed surgery group flies had lower activity indices than control flies (*P = *0.02; *U*-test with Holm–Bonferroni correction). *P < 0.05. **e** Flies that had surgery or glue on the cuticle had indistinguishable mean locomotor speeds from control flies on Days 6–20 (*P* = 0.1–0.9; Kruskal–Wallis ANOVA; *n* = 8–10 flies per group). On Days 2 and 30, the groups exhibited significant differences (*P* = 0.03, Day 2, *P *= 0.04, Day 30; Kruskal–Wallis ANOVA; *n* = 7–10 flies per group), which were insufficient to yield significant paired, post-hoc tests between groups (*P* = 0.05–0.6; U-test with Holm–Bonferroni correction). **f**, **g** Maximal walking speeds (**f**) and odor avoidance indices (**g**) were indistinguishable between groups for 30 days (*P* = 0.2–0.9 for all comparisons of walking speeds and odor avoidance indices; *n* = 7–10 flies per group; Kruskal–Wallis ANOVA). Error bars: s.e.m.

After surgery and their release from the silica fiber, flies seemed to behave normally and could even mate and produce fertile offspring. However, to perform more in-depth checks for any behavioral effects of the surgical procedures we examined locomotor and odor avoidance behaviors in individual female flies (Fig. 3a–c).

We first looked for potential short-term effects of surgery. Two days after surgery, the activity levels and walking speeds of flies in the two experimental groups and the control group were distinct (*P* = 0.03 for activity levels, *P *= 0.03 for walking speeds; *n* = 10 flies per group; Kruskal–Wallis analysis of variance (ANOVA); Fig. 3d, e). However, post-hoc analyses showed that, for both experimental groups, neither activity levels nor mean walking speeds differed significantly from values measured in control flies (*P* = 0.06–0.8 for activity, *P* = 0.05–0.6 for speeds; Mann–Whitney *U*-tests with Holm–Bonferroni correction). These inconclusive findings, with contrary results from the ANOVA and post-hoc analyses, suggest there are mild short-term effects of the gluing procedure, detectable only at the limits of our study’s statistical power. Notably, the surgical procedures caused no additional detectable short-term harm beyond that of the gluing step.

By 6 and 10 days post-surgery, flies in the surgery group regained normal activity levels and walking speeds (*P *= 0.6–0.9 for activity levels, *P* = 0.7–0.9 for speeds; *n* = 10 flies per group; Kruskal–Wallis ANOVA). Later on, at Days 20 and 30 after surgery, flies in the three groups again had detectably different activity levels and mean walking speeds (*P* = 0.02–0.04 for activity, *P* = 0.04–0.1 for speeds; *n* = 7–10 female flies; Kruskal–Wallis ANOVA). However, as with the short-term effects of surgery, post-hoc analyses revealed that mean speeds and activity levels of the individual experimental groups were statistically indistinguishable from those of the control group (*P* = 0.08–0.9 for activity,* P* = 0.09–0.2 for speeds; Mann–Whitney *U*-tests with Holm–Bonferroni correction; Fig. 3d, e). An exception arose on Day 30, when the activity level of the surgery group was detectably lower than that of control flies (*P* = 0.022; Mann–Whitney *U*-test with Holm–Bonferroni correction). Of note, flies in both the surgery and glue groups had normal peak walking speeds and odor avoidance behavior for 30 days (*P *= 0.2–0.9; Kruskal–Wallis ANOVA; Fig. 3f, g), indicating normal olfactory sensorimotor function. Overall, female flies were generally healthy for at least 30 days after surgery, despite some subtle effects on specific behavior traits that were barely detectable in statistical analyses of the data from a total of 105 flies.

### Time-lapse two-photon imaging of neural structure

**Fig. 4:**
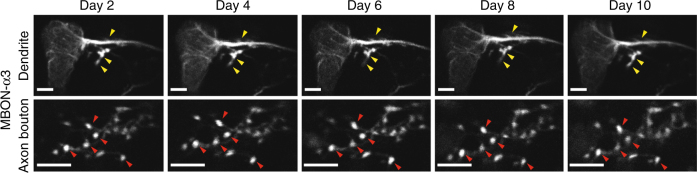
Long-term in vivo imaging of neural structure. Time-lapse two-photon imaging of the dendritic arbor (upper) and axonal boutons (lower) of a fluorescent mushroom body output neuron (MBON-α3) in a live fly (*MB093C-GAL4>20×UAS-6×GFP*). Major dendritic structures (yellow arrowheads) and axonal boutons (red arrowheads) were stable for 10 days. Scale bars: 10 μm. See also Supplementary Fig. 2 for additional time-lapse image datasets

As a first illustration of the applicability of our chronic fly preparation to time-lapse imaging studies, we combined it with two-photon microscopy to inspect the morphologies of identified, individual neurons across multiple days. Until now, it has not been possible to track neural structures in a longitudinal manner in the live adult fly brain. In flies expressing the green fluorescent protein (GFP) in the MBON-α3 mushroom body output neuron, we repeatedly imaged the dendritic arbors of this neuron over periods of 8–10 days (*n* = 4 female flies; fly ages were 3–4 days post-eclosion on the day of laser surgery; Supplementary Fig. 2). Across this time span, the axonal boutons, major dendrites, and translucency of the preparation all remained constant (Fig. 4), illustrating a capacity for time-lapse imaging of neural architecture at micron-scale resolution.

### Long-term imaging of odor-evoked neural Ca^2+^ activity

**Fig. 5:**
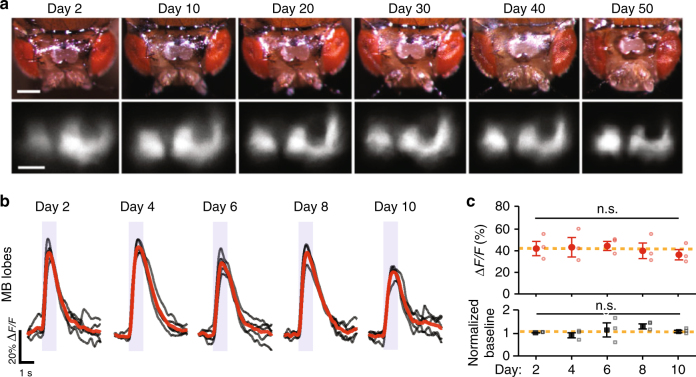
Long-term in vivo imaging of neural Ca^2+^ activity. **a** Example images of the surgically created imaging window (upper panels) and fluorescent mushroom bodies expressing the Ca^2+^ indicator GCaMP6f in *OK107-GAL4>20×UAS-GCaMP6f* flies (lower panels). The imaging window was transparent across the 50-day interval, and GCaMP6f expression appeared stable across this period. Scale bars are 200 µm (upper panels) and 100 µm (lower panels). **b** Example traces of odor-evoked neural Ca^2+^ activity in the mushroom body of an *OK107-GAL4>20×UAS-GCaMP6f* fly. Gray traces: individual trials (*n* = 5). Red traces: mean responses. Shaded intervals: periods of olfactory stimulation (1 s duration) with 2% ethyl acetate. **c** Peak amplitudes of odor-evoked Ca^2+^ activity (upper) and normalized levels of baseline fluorescence (lower) in the mushroom body were both stable for 10 days (*P* = 0.4 and 0.3 for upper and lower panels, respectively; Friedman ANOVA; *n* = 3 female *OK107-GAL4>20×UAS-GCaMP6f* flies). Closed data points denote mean values, and open data points mark values for individual flies. We normalized the baseline fluorescence levels in each fly to their values on Day 2. Error bars are s.e.m. Yellow dashed lines denote mean values averaged over all 10 days

Next, we performed long-term Ca^2+^ imaging experiments in flies that expressed the GCaMP6f Ca^2+^ indicator^7^ in mushroom body neurons (see Methods section). We measured odor-evoked Ca^2+^ responses in these neurons for up to 50 days over 18 imaging sessions. Throughout this period, cuticle windows stayed transparent and waveforms of the evoked Ca^2+^ transients remained consistent (Fig. 5a, b; Supplementary Fig. 3a–c). For the first 10 days, peak amplitudes of odor-evoked responses and baseline fluorescence levels were also both stable (*P* = 0.4 and 0.3, respectively; *n* = 3 female flies; Friedman ANOVA; Fig. 5c). After 12 days, peak amplitudes of the odor-evoked Ca^2+^ responses became more variable (Supplementary Fig. 3b, c). These variations may partially have arisen from fluctuations in baseline fluorescence levels and might reflect an intrinsic aspect of long-term fluorescence Ca^2+^ imaging (Supplementary Fig. 3d). Nonetheless, the time-dependent variations were relatively subtle, and the results demonstrate the feasibility of assessing neural Ca^2+^ dynamics over multiple weeks.

### Long-term voltage imaging of dopamine neuron dynamics

We next performed long-term optical imaging of neural activity in flies expressing a genetically encoded fluorescent voltage indicator. We chose the indicator Ace2N-2AA-mNeonGreen, which can report single action potentials with near 100% detection accuracy and ~200 µs timing precision in sparsely labeled neurons of the intact fly brain^8^.

**Fig. 6:**
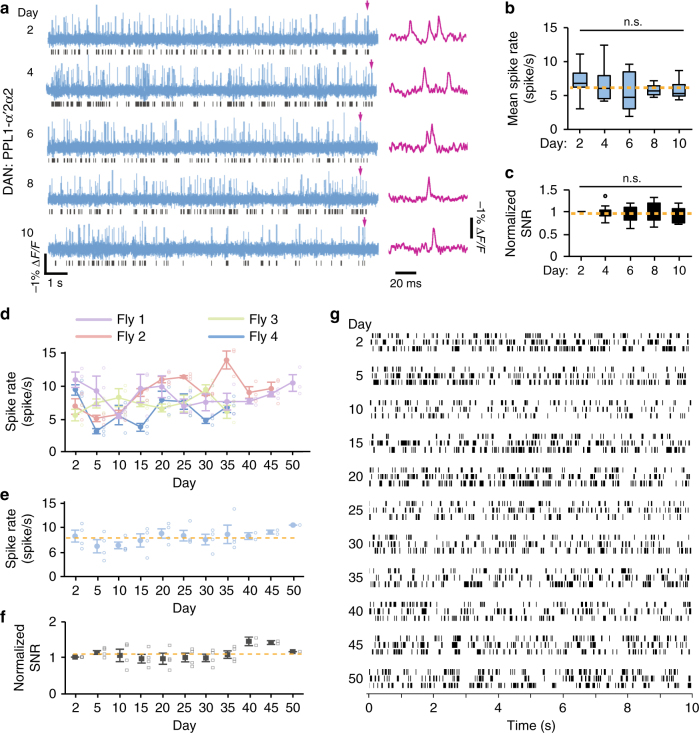
Imaging neural voltage dynamics for 30–50 days in live flies. **a** Traces (blue) of spontaneous activity of a PPL1-α’2α2 dopamine neuron expressing the Ace2N-2AA-mNeon voltage indicator in a *MB058B-GAL4>20×UAS-Ace2N-2AA-mNeon* fly, alongside magnified views (magenta) of individual spikes marked with arrows. Raster plots below each voltage trace show the corresponding spike trains. **b**,** c** Box-and-whisker plots showing that spontaneous spiking rates (**b**) and normalized signal-noise-ratios (SNRs) of optical spike detection (**c**) in PPL1-α’2α2 neurons were stable for 10 days (*P* = 0.7 and *P* = 0.2, respectively; *n* = 10 female flies; Friedman ANOVA). We defined SNR as the mean signal amplitude per spike divided by the s.d. of the baseline voltage signal. We normalized SNR values in each fly to the values measured on Day 2. For each fly, during each session we acquired three separate 10-s-recordings of spontaneous spiking. We averaged spiking rates and SNR values for each fly across these three recordings; using the resulting mean values we computed the box-and-whisker elements for each session. **d** Rates of spontaneous spiking by PPL1-α’2α2 neurons in four female *MB058B-GAL4>20×UAS-Ace2N-2AA-mNeon* flies. Open data points: values from individual 10-s-long recordings. Closed data points: mean values averaged across the three recordings per session performed for each fly. Only one fly survived 50 days.** e**,** f** Mean firing rates (**e**) and normalized signal-noise-ratios (SNR) (**f**) of spiking in PPL1-α’2α2 neurons for the same flies as in **d**. SNR values normalized in each fly to the values from Day 2. Closed data points: mean values averaged across the four flies. Open data points: values from individual flies. **g** Raster plots of spiking activity of a single PPL1-α’2α2 neuron in the fly that survived 50 days. Error bars in **d**–**f**: s.e.m. Yellow dashed lines: time-averaged mean values. In box-and-whisker plots, boxes cover the middle two quartiles, horizontal lines inside boxes denote median values, whiskers extend to 1.5 times the interquartile range, and outlier data points are shown individually

After expressing this indicator in the PPL1-α’2α2 dopaminergic neuron (see Methods section), we detected action potentials at single-spike resolution at the cell’s axonal region for up to 50 days (11 imaging sessions; 1 kHz imaging frame rates; Fig. 6a–g). Mean rates of spontaneous spiking were stable for the first 10 days (*P* = 0.7; *n* = 10 female flies; Friedman ANOVA; Fig. 6b). Signal-to-noise ratios of the detected spikes were also nearly stationary for the first 10 days (*P* = 0.2; *n* = 10 female flies; Friedman ANOVA; Fig. 6c). To estimate the fidelity of optical spike detection, we used a signal detection theoretic analysis established previously for quantitative performance evaluations of neural activity imaging^8, 9^. From this analysis, we determined that the spike detection fidelity, *d′*, had a value of ~7.1 at our 1 kHz image frame acquisition rate, which implies mathematically a spike detection error rate (including false positives and false negatives) of ~0.4/s. This estimated error rate is <7% of the mean rates of spontaneous spiking (~6–10/s) observed in PPL1-α’2α2 neurons.

Starting at 10-day post-surgery, long-term voltage imaging revealed daily fluctuations in the mean rates and signal-to-noise ratios (SNRs) of the detected spikes in individual and groups of flies (Fig. 6d–f). These observations were consistent with the daily fluctuations in baseline and peak Ca^2+^ transient amplitudes that arose in our Ca^2+^ imaging studies by 12 days after surgery (Supplementary Fig. 3b–d). As spike rate determinations require only spike detection, a binary assessment, rather than continuously valued estimates of fluorescence amplitudes, the fluctuations in spiking properties found with voltage imaging seem more likely to reflect physiological variations rather than changes in imaging quality (for which we saw no other evidence). Thus long-term voltage imaging provides a potent means of recording neural spiking dynamics at millisecond resolution over ≥10 days in live flies (Fig. 6g).

### Differential responses of dopamine cells to mechanical stress

As a further demonstration of our ability to follow subtle physiological changes, we tracked the spiking properties of two types of PPL1 dopaminergic neurons (PPL1-α’3 and PPL1-α’2α2) in response to prolonged mechanical stress, which is commonly used in fly biology studies as a means of negative reinforcement or sleep deprivation^10, 11^. These two types of dopamine neurons innervate different compartments of the mushroom body α/α’ lobes^12^ and have been implicated in the regulation of memory^13^, sleep^14^, and sensation^15^. Past studies have found that PPL1-α’2α2 but not PPL1-α’3 neurons contribute to the formation of both short- and long-term aversive memories^13^, whereas PPL1-α’3 but not PPL1-α’2α2 neurons have a wake-promoting function in sleep regulation^14^. However, it has remained unclear whether these two distinct types of PPL1 neurons have different long-term physiological responses to environmental influences.

**Fig. 7:**
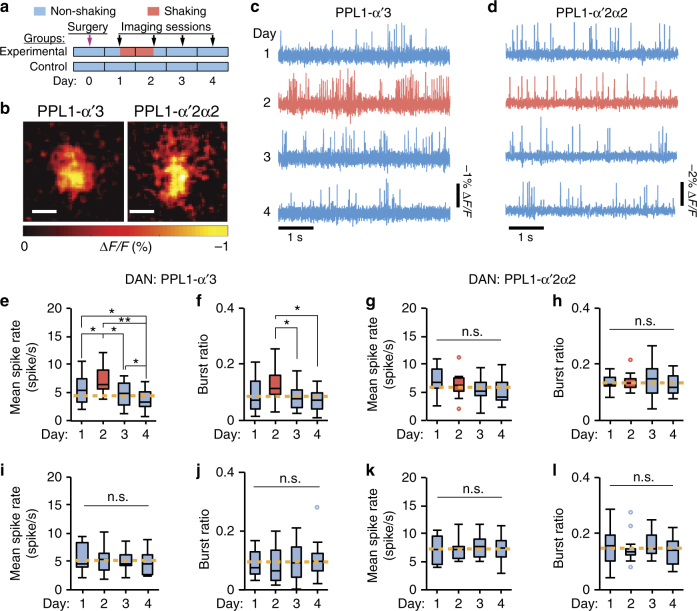
Spontaneous spiking responds differently to mechanical stress in PPL1-α‘3 versus PPL1-α‘2α2 dopaminergic neurons. **a** Timeline: Laser surgery on Day 0, then daily voltage imaging for 4 days. From noon on Day 1, experimental but not control flies underwent 24 h of ongoing shaking. We performed three separate 10-s recordings per session per fly. We averaged spiking metrics for each fly across these three recordings; using the resulting mean values, we computed the box-and-whisker elements in **e**–**l**. **b** Spatial maps of mean fluorescence response (Δ*F/F*) at the spike peak in axonal regions of a PPL1-α’3 neuron in a *MB304B-GAL4>20×UAS-Ace2N-2AA-mNeon* fly (left) and a PPL1-α’2α2 neuron in a *MB058B-GAL4>20×UAS-Ace2N-2AA-mNeon* fly (right), averaged over 140 and 113 spikes, respectively. Scale bars: 10 μm. **c** Activity traces of a PPL1-α’3 neuron, showing increased spiking after shaking (Day 2). **d** Activity traces of a PPL1-α’2α2 neuron, showing normal spiking after shaking. **e**,** f** Spiking rates (**e**) and burst ratios (**f**) of PPL1-α’3 neurons were elevated after shaking and then declined below baseline on Day 4 (*P* = 4 × 10^−4^ in **e**, *P *= 0.01 in **f**; *n* = 15 flies; Friedman ANOVA; *P < 0.05, **P < 0.01 post-hoc Wilcoxon signed-rank test with Holm–Bonferroni correction). We computed the burst ratio as the number of spikes in a cell’s activity record occurring <20 ms after the preceding spike, divided by the total number of spikes in the record. **g**,** h** Spiking rates (**g**) and burst ratios (**h**) of PPL1-α’2α2 neurons were unaffected by shaking (*P* = 0.05 in **g**, *P* = 0.9 in **h**; *n* = 13 flies; Friedman ANOVA). **i**,** j** In control flies, spiking rates (**i**) and burst ratios (**j**) of PPL1-α’3 neurons were stationary (*P* = 0.9 in **i**, *P* = 0.5 in **j**; *n* = 15 flies; Friedman ANOVA). **k**,** l** In control flies, spiking rates (**k**) and burst ratios (**l**) of PPL1-α’2α2 neurons were stationary (*P* = 0.8 in **k**, *P* = 0.3 in **l**; *n* = 13 female flies; Friedman ANOVA). Yellow dashed lines: mean values averaged across all days without shaking. Box-and-whisker plots are formatted as in Fig. 6

Using the Ace2N-2AA-mNeonGreen voltage indicator and our chronic preparation, we monitored the patterns of spontaneous spiking in the axonal regions of the two types of dopamine neurons in flies subject to a 4-day experimental protocol with daily imaging sessions (Fig. 7a, b; Supplementary Fig. 4a–l). After laser surgery, we gave the flies 1 day of rest, followed by a full day of mechanical shaking, and then another 2 days of rest.

After a day of mechanical stress, PPL1-α’3 neurons increased their rates of firing both single and bursts of action potentials (*P* = 4 × 10^–4^ and 0.01, respectively, *n* = 15 female flies; Friedman ANOVA), whereas these rates declined below baseline levels during the 2 recovery days (Fig. 7c, e, f; Supplementary Fig. 4c). By comparison, PPL1-α’2α2 neurons maintained consistent rates of firing single spikes and spike bursts for all 4 days (*P* = 0.05 and 0.9, respectively; *n* = 13 female flies; Friedman ANOVA) (Fig. 7d, g, h; Supplementary Fig. 4f). Control flies that did not undergo mechanical shaking exhibited none of the changes (Fig. 7i–l). Analyses of spike waveforms in flies that were shaken revealed no long-term changes in the action potential waveforms of either PPL1-α’3 or PPL1-α’2α2 neurons (Supplementary Fig. 4g–l), indicating the consistent quality of the long-term optical recordings.

## Discussion

To enable longitudinal imaging studies of the adult fly brain, we created a chronic fly preparation that allows repeated intravital imaging of neural structure and dynamics across unprecedented intervals up to 50 days in duration. In female flies, our technique caused minimal-to-no detectable harm to the animal’s basic health and behavior. In male flies, our procedures led to a statistically significant decline in lifespan. Notwithstanding, our approach allows time-lapse studies in male flies over tens of days, a major improvement over past approaches that were limited to <24 h of imaging in adult flies^5, 6^.

The survival curve data indicate that the increased mortality of male flies is probably not a direct consequence of the laser treatment per se but more likely arises from the handling and related stressors that are part of the entire surgical and time-lapse protocol. Simply applying glue to the male fly led to a statistical decrease in lifespan that was indistinguishable from that of the entire surgical protocol (Fig. 2b). Although there are undoubtedly multiple different stressors at different steps in the protocol, we suspect the glue itself may be a noteworthy contributor to fly mortality. After flies returned to a food vial (Fig. 1a), some deaths occurred when the glue caused flies to become stuck either to food or the vial wall. Flies that are young or female are stronger and may be more capable of escaping when this occurs than older or male flies. We suggest that future practitioners may be able to improve fly survival rates by applying the minimal amount of glue necessary.

Our time-lapse voltage imaging studies in dopamine neurons revealed the differential responses of PPL1-α’3 and PPL1-α’2α2 neurons to prolonged mechanical stress. Given that the activity of the PPL1-α’3 neuron can promote wakefulness^14^, the increases we found in its spontaneous spiking and spike bursting activity after prolonged mechanical shaking might serve to induce a higher level of arousal in the face of a stressful environment. After the termination of mechanical shaking, activity rates in the PPL1-α’3 neuron gradually declined back to and then below baseline levels, which might reflect a rebound stage of sleep recovery. Although our observational data do not permit functional or causal interpretations, the data do show that the spontaneous dynamics of the PPL1-α’3 neuron reflect the fly’s history of stress over the past couple days, even after an environmental stressor has been removed.

In addition to the optical techniques showcased here, our chronic fly preparation is compatible with a variety of recent optical imaging and optogenetic methods for use in head-fixed or freely moving flies^16–18^. By combining our approach with recently developed robotic methods for automated fly handling^19^, it might be possible in the future to incorporate long-term intravital imaging into large-scale genetic screens based on assessments of fly neurophysiological or behavioral traits. Given the powerful genetic toolkits that already exist for use in fruit flies, our preparation for long-term imaging immediately opens new opportunities to investigate the molecular and cellular mechanisms of how individual neurons respond over time to environmental influences, change across the adult fly lifespan, or alter their properties due to brain disease.

## Methods

### Fly stocks

We obtained *OK107-GAL4*, *R55D11-GAL4*, *20×UAS-GCaMP6f*, and *20×UAS-6×GFP* flies from the Bloomington Stock Center. The FlyLight Project Team at Janelia Research Campus kindly provided *MB304B-GAL4*, *MB093C-GAL4*, and *MB058B-GAL4* flies and the pJFRC7 vector plasmid. To create *20×UAS-Ace2N-2AA-mNeonGreen* flies, we sub-cloned Ace2N-2AA-mNeon cDNA into the pJFRC7 vector using its XbaI and XhoI restriction sites^20^ and added a *Drosophila* Kozak sequence (CAAA) prior to the start codon of Ace2N-2AA-mNeon. A commercial transformation service (BestGene Inc.) inserted the pJFRC7-Ace2N-2AA-mNeon plasmid into the phiC31 docking site attP40, identified successful transformants, and provided us homozygous *20×UAS-Ace2N-2AA-mNeonGreen* flies.

We performed all locomotion and imaging experiments using female flies (2–10-day old at the time of laser surgery). We raised flies on standard cornmeal agar media under a 12 h light/dark cycle at 25 °C and 50% relative humidity. To provide retinal to the *20×UAS-Ace2N-2AA-mNeonGreen* flies, we dissolved all trans-retinal powder in 95% ethanol as the 20 mM stock and diluted it with fly food to 400 µM. We collected adult female flies (1–3 days old) after eclosion and transferred them to the 400 µM retinal food for 6–8 days before surgery.

### Fly mounting

To mount flies in preparation for either laser surgery and/or in vivo imaging, we anesthetized the flies by placing them on ice for 1 min and then transferred them to the cooled surface (∼4 °C) of an aluminum thermoelectric cooling block (Supplementary Fig. 1a, b; Supplementary Movie 1). While viewing the fly though a dissection microscope (Leica MZ6) with a 40× objective lens, we adjusted the fly’s orientation using a dull pair of forceps (Dumont #55, Fine Science Tools) such that its ventral side contacted the aluminum block. We then secured a custom-made silicon fixture onto the mounting apparatus directly above the fly. The silicon fixture had a groove in which a 125-μm-diameter fused silica optical fiber (Nufern; PLMA-YDF-10/125) was held with Kapton tape (Techni-tool; KPT-1/2).

Using a multi-axis stage to manipulate the entire cooling block (Supplementary Fig. 1a, b), we brought the posterior side of the fly’s thorax into contact with the fiber. We applied ~1 µL of ultraviolet (UV) light curing epoxy (NOA 89, Norland) to the contact point between the fiber and the fly’s thorax and then cured the epoxy with UV light for 30 s. To minimize head motion, we glued the fly’s head to the thorax using UV epoxy. Generally, flies awoke and resumed activity ~1 min after mounting and were able to move their legs and wings. To minimize the side effects caused by anesthesia, we allowed flies to recover for 30 min before starting imaging experiments.

To release the fly, we used the sharp tip of a surgical scalpel blade to break off the entire piece of cured UV epoxy from the fly thorax. We then returned the fly to a food vial for future use. This release procedure did not seem to have any discernible impact on the fly’s wellbeing.

### Laser microsurgery

To create an imaging window on the fly head, we used a previously described laser microsurgery system^6^ based on a 193-nm-wavelength excimer laser (GamLaser; EX5 ArF). Using a variety of pupil masks and 10-ns laser pulses, this system can cut holes in the fly cuticle with a range of diameters (~12–500 µm). After transferring a mounted fly to the surgery station, we created an optical window in the cuticle by laser drilling a 150-μm-diameter hole (30 pulses, delivered at 100 Hz, 36 µJ per pulse as measured at the specimen plane). This microsurgical procedure generally removed the cuticle, air sacs, and fat bodies and thereby exposed the underlying fluorescent cells **(**Fig. 1a, b). However, due to variations in head size and fly age, occasionally further rounds of laser dissection or manual cleaning of the cuticle were needed to make visible the underlying fluorescent cells. The brains of older flies (>20-day post-eclosion) usually have a greater number of fat bodies and less hemolymph as compared to younger flies, so in older flies we commonly needed additional laser pulses to cut through the cuticle as well as additional manual cleaning after surgery.

Immediately after surgery, we applied 1 µL of UV epoxy (NOA 68, Norland; 1.54 refractive index; ~99% optical transmission for wavelengths between 420 and 1000 nm) and cured it for 30 s to seal the cuticle opening. To maintain ~60% humidity surrounding the fly during the resealing process, we used a desktop ultrasonic humidifier (Air-O-Swiss; AOS 7146) to puff humidified air directly onto the fly while it was under the dissection microscope. After sealing the opening, we released the mounted fly from the fiber as described above and returned the fly to a food vial for later usage.

Overall, for each fly we required approximately ~3 min for mounting, ~1.5 min for laser surgery, and ~1.5 min for resealing of the cuticle. Some additional time was required for transferring flies, yielding an overall work speed of approximately eight flies per hour.

### Survival assay

To test the potential effects of laser microsurgery on fly viability, we measured the lifespans of three different groups of flies. The control group underwent no surgical treatments. The glue group received the UV glue atop the cuticle but no surgery. The surgery group underwent laser surgery and cuticle gluing. Each group comprised 25 male and 25 female flies. After the various treatments, we placed the individual flies into separate food vials. We recorded daily the number of living flies and transferred them to fresh food vials every 5 days. We kept the flies at 25 °C and 50% relative humidity, with a 12-h light/dark cycle.

### Locomotion and odor avoidance assay

For testing locomotor and olfactory behavior, we used a single-fly assay described previously^21^. This assay was performed during the flies’ light cycle within a printed plastic chamber (50 mm in length, 4 mm in width, and 1.5 mm in height). The floor and ceiling of the chamber were each covered by a glass coverslip (22 × 60 mm, No. 1, VWR). An additional layer of Chromatography paper (3 MM CHR, Whatman^TM^, GE Healthcare) was on the floor inside the chamber, allowing the flies to walk smoothly without slipping on the coverslip. Air or odor entered the chamber through two inlets (0.75 mm in width) at its left and right ends and exited through two outlets (0.75 mm in width) at its midpoint (Fig. 3a). The chamber was illuminated from below by an infrared (IR) light-emitting-diode (940 nm, EnvironmentalLights). We monitored the behavior of the fly in the chamber using a monochrome board camera operating at 30 fps and equipped with an IR-transmitting lens (DMM 22BUC03-ML, Imaging Source).

A custom-built olfactometer delivered a constant flow of air (100 mL/min) and switched between clean air (filtered using mineral oil) and odor-containing air (odorants dissolved in mineral oil). After transferring a fly into the behavioral chamber, we first delivered air into both sides of the chamber for 1 min and then delivered 3% 4-methyl-cyclohexanol (MCH) into the left side for 1 min. Afterward, we delivered air again to both sides for 30 s and then 3% MCH into the right side for 1 min (Fig. 3c).

### Fly tracking and data analysis

To computationally track the movements of a fly in the behavioral videos, we performed an image analysis with the following four main steps (Fig. 3b). (1) We drew a bounding box around the behavior track, cropping the image to include only the region-of-interest (ROI); (2) We created an image of the track background by averaging over all frames in the video. The algorithm then subtracted the background image from each individual frame; (3) We binarized the background-subtracted image using Otsu’s method, turning the background black and foreground objects white. After a series of erosion and dilation operations on the resulting black/white image, we eliminated all white objects <50 pixels in size (noise), leaving only the white object corresponding to the fly; (4) We calculated the centroid position of this object and stored it in memory as the fly’s current location. To calculate the walking speed of the fly, we multiplied the distance between its successive centroid positions and the camera frame rate.

We classified the fly’s behavior into active states (>1 mm/s walking speed) and stationary states (<1 mm/s speed). We calculated the activity index as the fraction of the total session that the fly spent in an active state. For calculations of mean walking speed, we excluded from analysis all times when the fly was stationary. To calculate the odor avoidance index, we computed the ratio of the total time that the fly spent on the side of the chamber where the odor entered to that spent on the side with clean air (Fig. 3c).

### Two-photon microscopy

After mounting the fly on its thorax using a silica fiber, we put a coverslip (22 × 22 mm^2^, No. 0, Electron Microscope Sciences) above the fly’s head. On the top side of the coverslip, we glued a stainless steel washer (Belleville) to contain the water (~100 µL) used with a water-immersion objective lens (Supplementary Fig. 1b). We also placed a small drop (~1 µL) of water between the coverslip and the fly cuticle (Supplementary Fig. 1c). We used a two-photon microscope (Prairie Technologies) equipped with a 1.0 NA, 20× water-immersion objective lens (XLUMPlanFL, Olympus). We imaged the neurons at a frame rate of 0.6 Hz (1024 × 1024 pixels) using ultra-short pulsed illumination of 920 nm, with 10–15 mW of power at the specimen plane.

### Calcium imaging

For imaging odor-evoked Ca^2+^ dynamics in the mushroom body lobe, we used a custom-built upright epi-fluorescence microscope equipped with a 0.4 NA, 20× air objective lens (PLN20X, Olympus). We used a 475/50 nm excitation filter (BrightLine), a 488 nm dichroic (Semrock), and a 534/30 nm emission filter (BrightLine). We illuminated the sample using the 480-nm wavelength module of a solid-state light source (Heliophor, 89 North) with 5–10 mW of power at the specimen plane. We acquired images at 50 Hz, using a scientific-grade CMOS camera (Zyla 4.2, Andor) to sample image regions of interest with 2 × 2 pixel binning.

On each day, we presented five trials of 2% ethyl acetate to each fly through a glass capillary (0.35 mm inner diameter) ~1 mm in front of the fly’s antenna. Odor delivery lasted for 1 s and was followed by clean air for 19 s. To analyze the Ca^2+^ activity traces, we corrected the raw Ca^2+^ videos for any brain movement by using the Turboreg algorithm for image registration^22^ (http://bigwww.epfl.ch/thevenaz/turboreg/). We then selected pixels whose mean fluorescence intensity was in the top 10% of all pixels; we defined their union as the ROI over which we computed spatially averaged, time-dependent changes in relative fluorescence intensity, Δ*F*(*t*)*/F*_0_, where *F*_0_ was the mean fluorescence in the ROI averaged over the entire video. We corrected for photobleaching by fitting a double exponential function to the mean fluorescence trace, *F*_0_, and then normalizing *F*_0_ by the fitted double exponential trace.

### Voltage imaging

To image voltage dynamics, we used a 1.0 NA, 20× water-immersion microscope objective lens (XLUMPlanFL, Olympus) and the same custom epi-fluorescence microscope as for Ca^2+^ imaging. We used a 503/20 nm excitation filter (Chroma), a 518 nm dichroic (Chroma), and a 534/30 nm emission filter (BrightLine). We illuminated the sample using the 500-nm wavelength module of the solid-state light source (Heliophor, 89 North) with 5–8 mW of optical power at the specimen plane. We acquired images at 1 kHz, using the scientific-grade camera and 2 × 2 pixel binning. For each fly, during each imaging session we sampled spontaneous neural activity during three separate recording trials, each 10 s in duration.

To analyze the voltage traces, we used the same procedures described above for Ca^2+^ imaging to correct for brain motion, identify an ROI, extract a Δ*F*(*t*)*/F*_0_ trace, and correct for photobleaching. To identify individual action potentials, we high-pass filtered the Δ*F*(*t*)*/F*_0_ trace by subtracting a median-filtered (40-ms window) version of the trace and then identified as spikes the local peaks with amplitudes >3.5 s.d. of the mean baseline fluorescence^8^. We determined the SNR as the mean amplitude of the spikes divided by the mean value of the baseline fluctuations (1 s.d.) in fluorescence. We defined the burst ratio as the fraction of spikes with an interspike interval to the preceding spike of <20 ms. For each fly, we averaged each of these spiking activity metrics across the three 10-s recordings performed in each session; using the resulting mean values, we computed the box-and-whisker plots in Figs. 6 and 7 and Supplementary Fig. 4.

To analyze spike waveforms, we determined the mean waveform by averaging over all spikes within a trial. We then performed a spline interpolation (10-µs intervals) of the mean waveform and from the resultant determined the spike amplitude and full-width half-maximum value of the spike duration. We used a signal detection framework^8, 9^ to compute the spike detection fidelity, *d′*, in a way that takes into account both the duration and intensity of the fluorescence waveform in response to an action potential. We estimated the photon shot noise in the optical voltage signal as the standard deviation of the fluctuations in baseline fluorescence. We then computed *d’* using the empirically determined, mean optical spike waveform (see equations S1–S4 of ref. ^9^).

### Mechanical stress

Before experiments on mechanical stress, we separated the flies into individual food vials and allowed them to rest for 1 day. Then we put the vials on a compact digital mini-rotator (Thermo Scientific) with a rotating speed of 200 rpm for 1 day. Two more days of rest followed (Fig. 7a).

### Statistical analyses

We performed all statistical analyses using MATLAB (Mathworks) software. Sample sizes were chosen using our own and published empirical measurements to gauge effect magnitudes. We did not use a formal randomization procedure. However, at the start of each experiment we randomly selected flies in an informal manner from a larger group of flies that were raised together. We then performed surgery on the selected flies and used these same flies for all subsequent sessions within a given longitudinal experiment. Experimenters were not blind to each fly’s genotype. For statistical testing, we used the non-parametric Kruskal–Wallis ANOVA and Friedman ANOVA to avoid assumptions of normal distributions or equal variance across groups. For post-hoc pairwise comparisons, we used two-sided versions of Mann–Whitney *U*-test or Wilcoxon signed-rank test with a Holm–Bonferroni correction for multiple comparisons. In box-and-whisker plots, the boxes cover the middle two quartiles of each dataset, horizontal lines inside the boxes indicate median values, whiskers extend to 1.5 times the interquartile range, and individual points indicate outliers.

### Data availability

Readers interested in either the experimental data or the software code used for analyses should please contact the corresponding authors.

## Electronic supplementary material

Supplementary Information

Description of Additional Supplementary Files

Supplementary Movie 1
