# Supplementary material for: Long-term optical brain imaging in live adult fruit flies
Source: Nat Commun. 2018 Feb 28;9:872. doi: 10.1038/s41467-018-02873-1 (PMC5830414; doi:10.1038/s41467-018-02873-1)
Supplement: Supplementary file 1 — Supplementary Information [file 41467_2018_2873_MOESM1_ESM.pdf]

Supplementary Figure 1 | High-resolution optical microscopy in live flies.

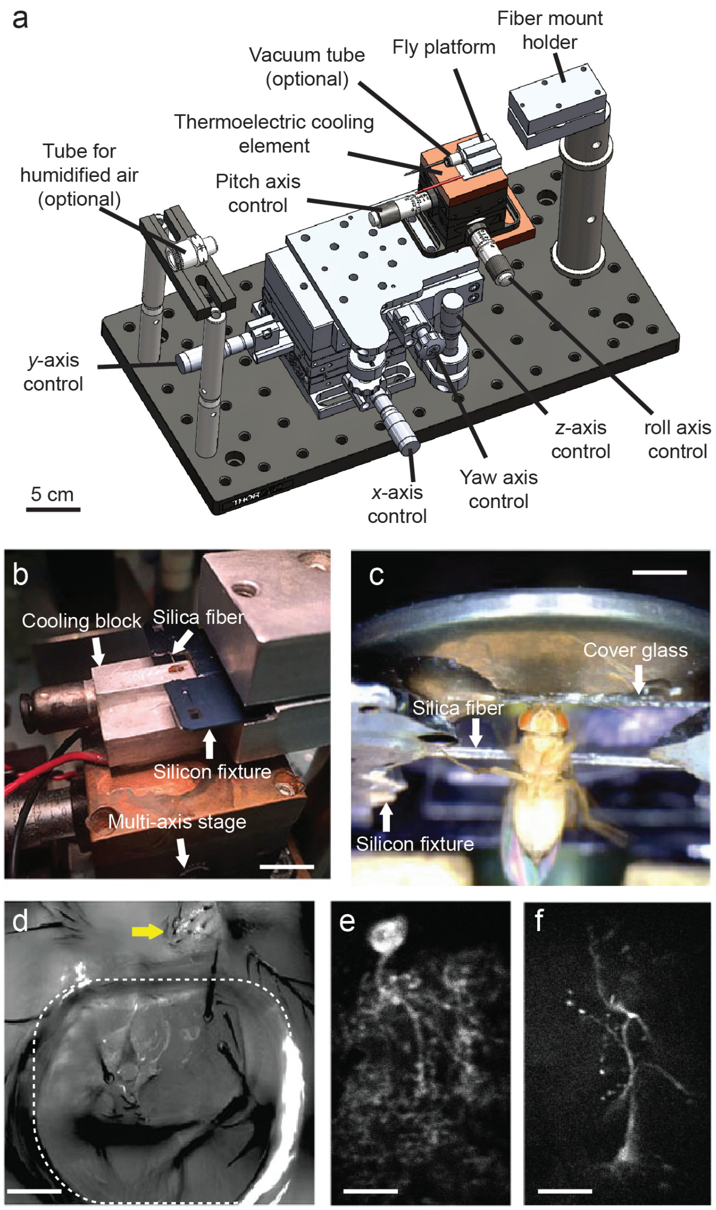

(a) Mechanical schematic of the fly mounting and manipulation station. To mount a fly, we first placed a silicon fixture holding a silica fiber into the fiber mount holder (see photograph in **b**). We then placed a fly onto the fly platform, where a thermoelectric cooling element chilled and thus anesthetized the fly. To attach the fly to the fiber, we brought the fly into contact with the fiber by adjusting the fly's position and orientation using a three-dimensional translation stage, a

dual-axis goniometer and a tip-tilt stage, which collectively provided six degrees of mechanical freedom. Once a fly was in contact with the silica fiber, we glued the fly to the fiber using ultraviolet-light curing glue (**Supplementary Video 1**). A vacuum suction line provided the additional option of using vacuum forces to manipulate the fly's body during positioning, if desired. The mounting station also had a tube for blowing humidified air across the fly.

(b) Photograph of the mounting apparatus, showing a fly that is resting on the chilled surface of the aluminum thermoelectric cooling block and positioned beneath the silica fiber.

(c) Photograph of a fly held to the silicon fixture via a silica fiber glued to its thorax. To hold a cover glass on the cuticle, we placed a drop (<1 μL) of distilled water between the fly's head and the cover glass. The resulting surface tension held the cover glass in place.

(d) An optical bright-field image of a fly's head, showing the distilled water (enclosed within the white dashed box) used to hold the cover glass on the fly's head. The fly's antennae (yellow arrow) did not contact the water and could still detect airborne odors.

(e) Two-photon fluorescence image of an olfactory local neuron in a female *R55D11-GAL4>20×UAS-6×GFP fly*, acquired through the surgically created imaging window.

(f) Two-photon image of a MBON-α3 neuron in a female *MB093C-GAL4>20×UAS-6×GFP* fly, acquired through the imaging window.

Scale bars are 5 cm in **a**, 1 cm in **b**, 2 mm in **c**, 100 μm in **d**, and 20 μm in **e** and **f**.

Supplementary Figure 2 | Long-term imaging of dendritic arbors in MBON-α3 neurons.

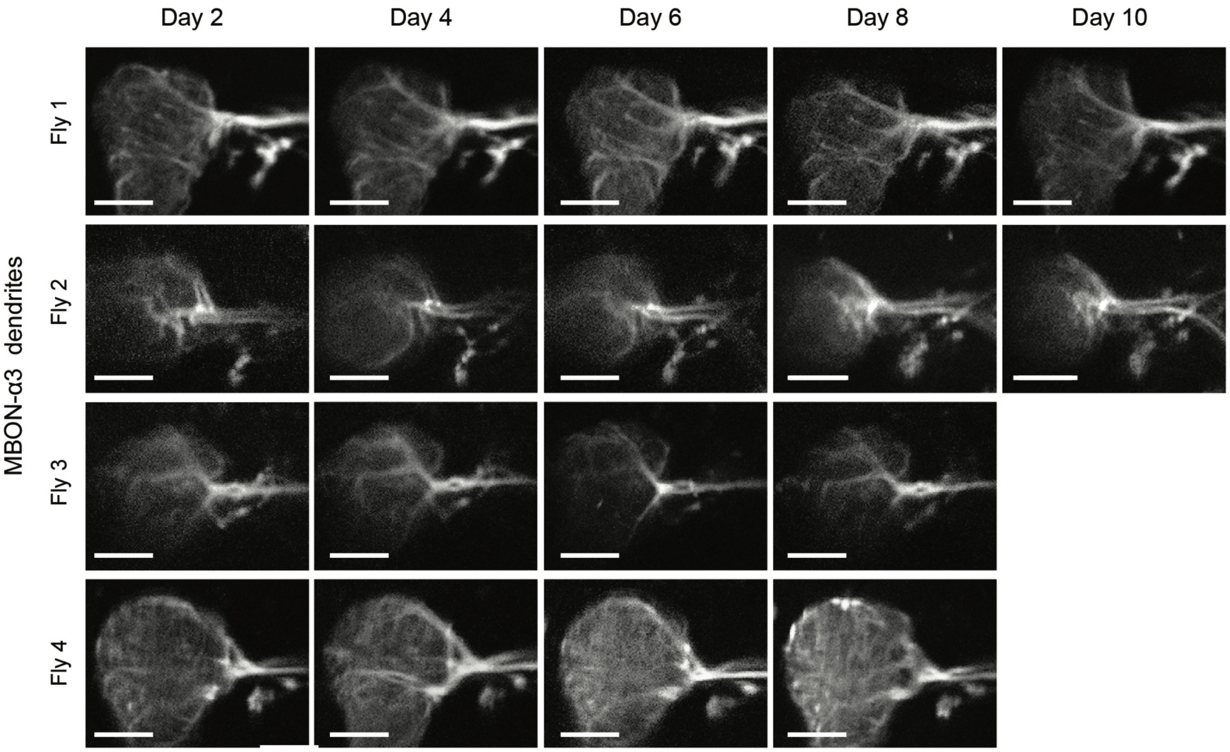

Time-lapse two-photon image datasets, showing the dendritic trees of the MBON-α3 mushroom body output neuron in four different live adult flies (*MB093C-GAL4>20×UAS-6×GFP*). We performed laser surgery on the flies when they were 3–4 days old. Two of the flies subsequently survived for 10 days, and the other two survived for 8 days. Scale bars: 10 μm.

Supplementary Figure 3 | Imaging odor-evoked Ca^{2+} activity for 50 days in the mushroom body of live flies.

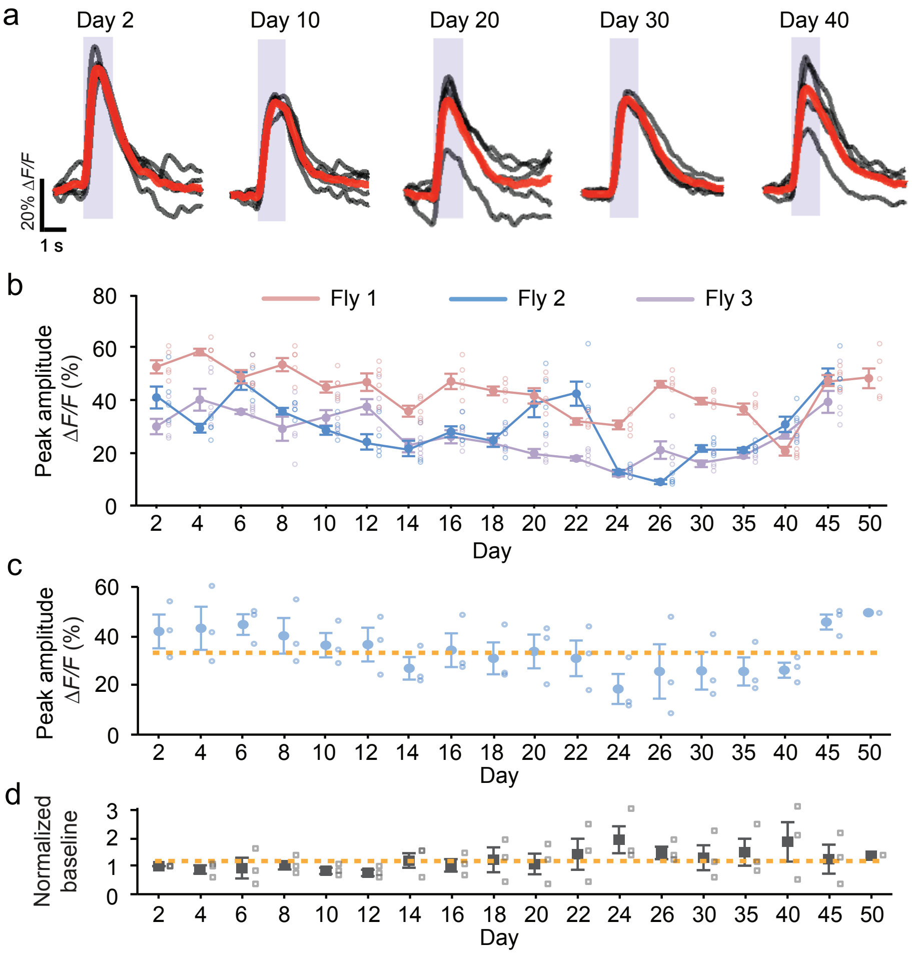

(a) Example traces of odor evoked Ca^{2+} activity in the mushroom body of an *OK107-GAL4>20×UAS-GCaMP6f* fly. Shaded intervals mark periods of olfactory stimulation (1 s in duration) with 2% ethyl acetate. Gray traces show the evoked responses on five different trials per day in the same fly. Red traces are the mean responses.

(b) Peak amplitudes of odor-evoked activity in the mushroom bodies of three individual flies (n = 5 trials per fly). Open data points mark values from individual trials of odor stimulation. Closed

data points denote mean values. Note that one fly survived 50 days, and two flies survived for 45 days.

(c) Mean peak amplitudes of odor-evoked activity for the same three flies as in **c**. Open circles mark the values determined from individual flies. Closed circles denote mean values averaged across all three flies.

(d) Normalized levels of baseline fluorescence from the same three flies as in **b** and **c**. We normalized the baseline fluorescence values from each fly to their measured values on Day 2. Open squares mark the values determined from individual flies. Closed squares denote mean values averaged across all three flies.

Error bars: s.e.m. Yellow dashed lines denote mean values averaged across 50 days.

Supplementary Figure 4 | The spontaneous spiking dynamics of PPL1-α’3 and PPL1-α’2α2 dopamine neurons are differentially regulated by mechanical stress.

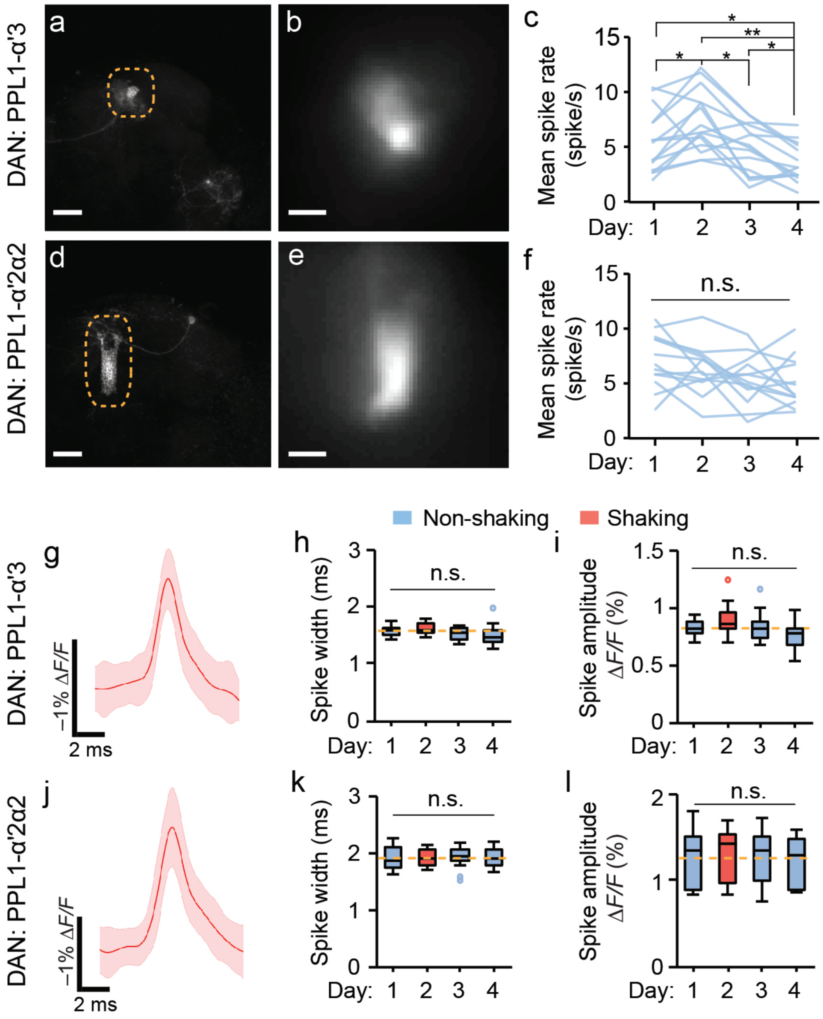

(a, b) A two-photon fluorescence image, **a**, and a one-photon fluorescence image, **b**, of a PPL1-α’3 neuron in a female *MB304B-GAL4>20×UAS-Ace2N-2AA-mNeon* fly. Yellow dashed box outlines the axonal region. Scale bars: 20 μm in **a** and 10 μm in **b**.

(c) Mean spiking rates of PPL1-α’3 neurons increased after mechanical stress (Day 2) and then declined on Day 4 ($P = 3 \cdot 10^{-4}$, n = 15 female flies; Friedman ANOVA). * and ** respectively denote post-hoc adjusted *P*-values of <0.05 and <0.01 from pairwise Wilcoxon signed-rank tests with Holm-Bonferroni correction. Each blue line shows the data from an individual fly.

(d, e) A two-photon fluorescence image, **d**, and a one-photon fluorescence image, **e**, of a PPL1-α'2α2 neuron in a female *MB058B-GAL4>20×UAS-Ace2N-2AA-mNeon* fly. Yellow dashed box outlines the axonal region. Scale bars: 20 μm in **d** and 10 μm in **e**.

(f) Mean spiking rates of PPL1-α'2α2 neurons were unaffected by mechanical stress (P = 0.05, n = 13 female flies; Friedman ANOVA). Each blue line shows the data from an individual fly.

(g) Mean optical waveform of spontaneously fired spikes (red trace) in the same PPL1-α'3 neuron as in **a** and **b**, averaged over 140 spikes. Red shading denotes s.d.

(h) Box-and-whisker plot showing that the mean durations (FWHM) of spontaneously fired action potentials in PPL1-α'3 neurons were unaffected by mechanical stress (P = 0.3; n = 15 female flies; Friedman ANOVA).

(i) Box-and-whisker plot showing that the mean amplitudes ($\Delta F/F$) of spontaneously fired action potentials in PPL1-α'3 neurons were unaffected by mechanical stress (P = 0.06; n = 15 female flies; Friedman ANOVA).

(j) Mean optical waveform of spontaneously fired spikes (red trace) in the same PPL1-α'2α2 neuron as in **d** and **e**, averaged over 113 spikes. Red shading denotes s.d.

(k) Box-and-whisker plot showing that the mean durations (FWHM) of spikes in PPL1-α'2α2 neurons were unaffected by mechanical stress (P = 0.5; n = 13 female flies; Friedman ANOVA).

(l) Box-and-whisker plot showing that the mean amplitudes ($\Delta F/F$) of spontaneously fired spikes in PPL1-α'2α2 neurons were unaffected by mechanical stress (P = 0.07; n = 13 female flies; Friedman ANOVA).

Yellow dashed lines indicate mean values averaged across all days without mechanical stress. In the box-and-whisker plots, boxes cover the middle two quartiles, horizontal lines inside the boxes denote median values, whiskers extend to 1.5 times the interquartile range, and outlier data points are shown individually.
