# Supplementary material for: Long-term optical brain imaging in live adult fruit flies
Source: Nat Commun. 2018 Feb 28;9:872. doi: 10.1038/s41467-018-02873-1 (PMC5830414; doi:10.1038/s41467-018-02873-1)
Supplement: Supplementary file 2 — Description of Additional Supplementary Files [file 41467_2018_2873_MOESM2_ESM.pdf]

## Description of Additional Supplementary Files

File Name: Supplementary Movie 1
Description:  Mounting and releasing a fly for a surgery or imaging session
